# Preclinical trial of the effectiveness of a safety nasogastric tube to detect the tube position based on tidal volume and pepsin assay results in the gastrointestinal tract of *Macaca fascicularis*

**DOI:** 10.1186/s12938-023-01128-5

**Published:** 2023-07-13

**Authors:** Sigit Mohammad Nuzul, Neng Tine Kartinah, Busjra M. Nur, Ermita I. Ibrahim Ilyas

**Affiliations:** 1 Master’s Program in Biomedical Science, Faculty of Medicine, Universitas Indonesia, Depok, Indonesia; 2 Physiology Department, Faculty of Medicine, Universitas Indonesia, Depok, Indonesia; 3 Physiology Department, Faculty of Medicine and Health, Universitas Muhammadiyah, Jakarta, Indonesia

**Keywords:** Nasogastric tube, Malposition, Pepsin

## Abstract

**Background:**

Tube misplacement into the tracheobronchial tract is associated with pneumothorax in 0.5% of cases. NGT verification only detects the position of the tube at the end of the procedure. Therefore, a safe nasogastric tube (SNGT) was created to detect the NGT position in real time in a simple and inexpensive way. This study aimed to prove the effectiveness of the SNGT prototype in *Macaca fascicularis*.

**Result:**

An SNGT producing 50% of the TV had 100% sensitivity and specificity in detecting the position of the tube at 100% of the TV, with a sensitivity of 100% and a specificity of 87.5%. There was a significant difference between the movement of the SNGT 50% TV and SNGT 100% TV airbags (*p* ≤ 0.05). However, there was no significant difference between the accuracy of placement of the 50% TV SNGT, 100% TV SNGT, and conventional NGT (*p* > 0.05). The pepsin enzyme had better sensitivity (100%) than pH paper (91.66%) in detecting the end-of-procedure tube position. This research has the potential to advance into human clinical trials.

**Conclusion:**

SNGTs are highly effective in detecting the NGT position inside the respiratory and digestive tracts to prevent misplacement.

## Background

Currently, NGTs are used for feeding, drug administration, gastric decompression, or gastric irrigation [1]. Most tubes are inserted blindly [2] through one naris and passed into the pharynx. When a patient can swallow, this aids entry into the esophagus and stomach. However, particularly in patients with depressed consciousness levels, the tube may enter the respiratory tract and cause trauma prior to discovery by end-of-procedure tube position checks [3].

Several kinds of conventional verification tests have been used to confirm the position of an NGT inside the stomach. The auscultation test (“whoosh test”) is performed via a rapid injection of air into the NGT tube while auscultating (listening to a “whoosh” sound) under the xiphoid process, but this test is inaccurate [4, 5]. The common first-line verification method is aspiration of fluid from the NGT tube to check if the pH is ≤ 5. However, both the respiratory and gastrointestinal tracts can have high pH values (> 6), especially if the patient is receiving acid-suppressing drugs [6].

Recent NGT verification methods include the use of pepsin, trypsin, and pH levels to confirm NGT placement and check for complications involving reflux of gastric fluid into the respiratory tract. Research was conducted using pH ≤ 6, pepsin ≥ 100 μg/ml, and trypsin ≤ 30 μg/ml as criteria for proper tube placement to precisely predict NGT placement. The results indicated that these three variables can correctly classify 93.4% of tubes positioned in the stomach [7].

Tube misplacement into the tracheobronchial tract is associated with pneumothorax in 0.5% of cases [8]. Ninety-seven percent of lung trauma is caused during placement and cannot be prevented by end-of-procedure position checks. To date, National Patient Safety Agency (NPSA, 2005, 2011, 2013; NHSI, 2016) alerts have only given guidance on how to prevent the 3% of lung trauma cases caused by placements undetected at the end of the procedure [9, 10].

The safety NGT (SNGT) is intended to preempt lung trauma. It is shaped like a normal NGT tube but has an airbag that can be expanded and collapsed when the tube is passed through the respiratory tract. However, how much air from the vital capacity of the lungs can enter the airbag and how long a tube will be inserted to reach the upper laryngeal area is unknown. An SNGT prototype has been made and patented (No. IDP000056746) [11]. The aim of this study was to prove the effectiveness of the SNGT prototype in NGT placement in the gastrointestinal tract and in preventing misplacement of the tube in the respiratory tracts of monkeys as experimental animals prior to human trials. Monkeys were chosen as test animals because of their similarity to humans in terms of anatomical structure and physiology [12].

## Results

This study was conducted from January to June 2021. Spirometric examination was performed in two animals from the SNGT group.

### Tidal volume

The TV was measured using a spirometer. The volume of the airbag in a recent study was created according to the TV of monkeys, as shown in Table 1. The airbag size of 100% of the TV was 6.5 × 5 × 1.6 cm, with a total volume of 52 ml. The airbag size of 50% of the TV was 4 × 5 × 1.25 cm, with a total volume of 25 ml.

**Table 1:** Tidal volumes of the SNGT groups

No	ID No	VE (ml)	RR	TV (ml)
1	140405	1,480	28	52.8
2	151220	1,050	21	50

### Effectiveness of the SNGT

On the basis of the results of the study, the Thornier-Remain confirmatory test was performed to determine the sensitivity and specificity of the SNGT 50% TV or SNGT 100% TV. The test calculations were performed using a 2 × 2 table to compare the results of the SNGT airbag verification with the laryngoscopic verification. The SNGT was correctly inserted into the digestive tract if there was no movement of the airbag. Movement of the SNGT airbag (inflation and deflation) indicated that the tube was misplaced in the respiratory tract.

Table 2 shows that the sensitivity and specificity of the SNGT 50% TV were both 100%. However, the sensitivity of the SNGT 100% TV was 87.5%, and its specificity was 100%. A decrease in sensitivity due to one of the insertions indicated movement of the airbag in the stomach.

**Table 2:** Differences between the SNGT 50% TV and SNGT 100% TV using a confirmatory test for detecting the tube position

No	Tube	Airbag movement	Laryngoscopic examination		Thornier-Remain	
			Gastrointestinal tract	Respiratory tract	Sensitivity (%)	Specificity (%)
1	SNGT 50% TV	Absent	8	0	100	100
		Present	0	8		
2	SNGT 100% TV	Absent	7	0	87.5	100
		Present	1	8		

**Fig. 1:**
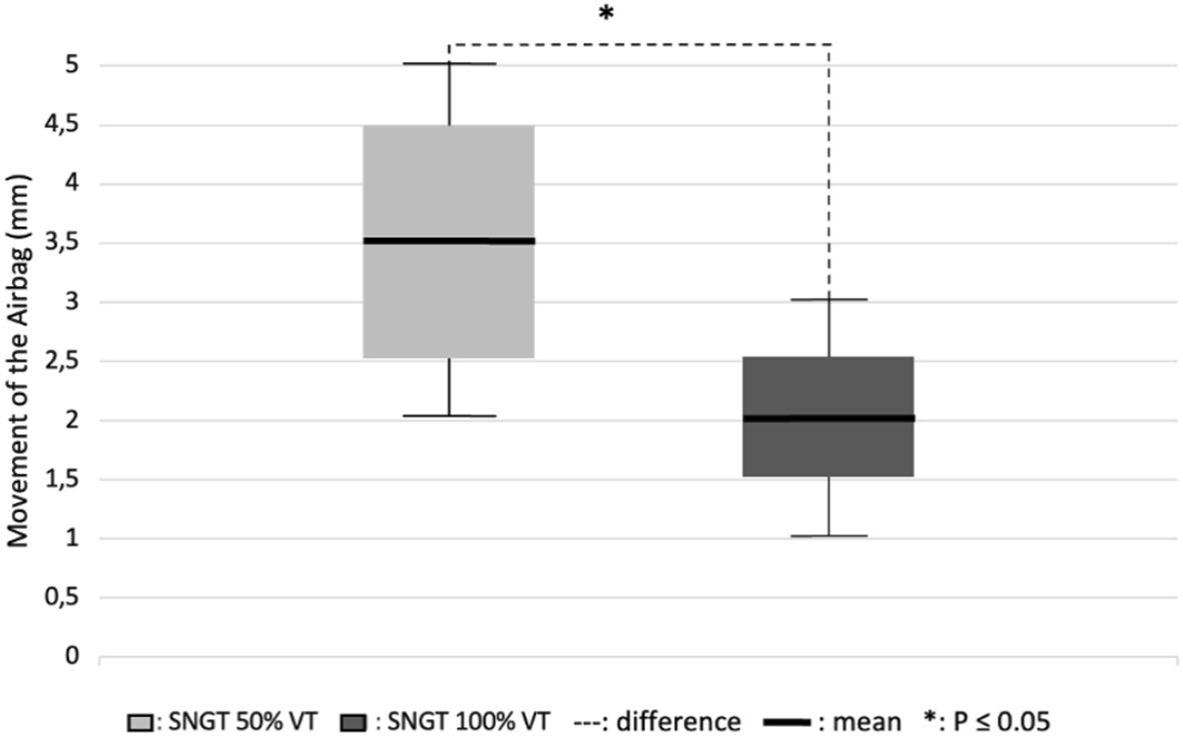
Movement of the airbags of the SNGT 50% and SNGT 100% TV

The results presented in Fig. 1 show that the mean movement of the airbag was 3.5 mm for the SNGT 50% TV and 2 mm for the SNGT 100% TV. The results of the *t-*dependent test showed a p-value of 0.026 (≤ 0.05), which indicates a significant difference.

**Fig. 2:**
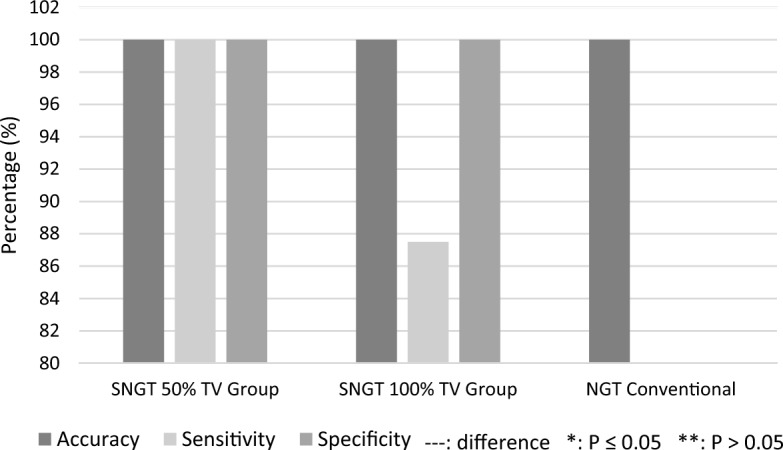
Accuracies of the SNGT 50% TV, SNGT 100% TV, and conventional NGT

The results presented in Fig. 2 show no significant differences in tube placement accuracy between the SNGT 50% TV, SNGT 100% TV, and conventional NGT. All tubes were inserted into the stomach on the first attempt. The sensitivity of the SNGT 50% TV was higher than that of the SNGT 100% TV in detecting the correct position of the tube in the gastrointestinal tract. Both the SNGT 50% TV and SNGT 100% TV had 100% specificity in detecting the position of the tube in the upper respiratory tract.

### Differences in the pH paper and pepsin enzyme assay

**Fig. 3:**
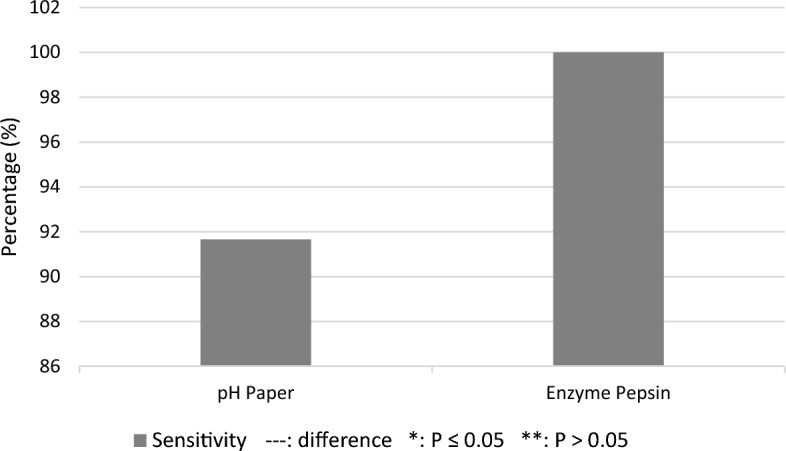
Differences in sensitivity between the pH paper and pepsin assays

The Thornier-Remain confirmatory test was performed to determine the sensitivity of the pH paper and pepsin assays. Figure 3 shows that the pepsin assay had a higher sensitivity (100%) than the pH paper (91.66%) in detecting the end position of the tube. Two samples in the pH test had false-positive results.

## Discussion

Using airbag movement at the base of the gastric tube is a new way to check for tube misplacement into the respiratory tract during placement. Respiratory misplacement is indicated by airbag movement due to air flow into the SNGT. To facilitate air flow, the SNGT has more side orifices (8–10 holes) than a traditional NGT (4–6 holes), allowing the airbag at the SNGT luer to expand during expiration and collapse during inspiration. These movements supposedly occur until the tube reaches the hypopharynx. Once the end of the tube is more deeply advanced (> 5 cm), two possible events will occur. First, the airbag will stop moving when the end of the tube enters the esophagus properly. Second, the airbag will continue to move and deflate when the end of the tube enters the trachea. If this happens, the tube must be withdrawn before going deeper and causing trauma, which would occur if only using an end-of-procedure check.

The results of this study are supported by several other studies that have taken advantage of the differences in physiological aspects between the respiratory and digestive tracts. According to Fan et al. [7], the method of verifying the NGT position can be performed with capnography/colorimetric capnometry [13]. According to Chau et al. [14], colorimetric devices are as accurate as capnography in detecting CO2 levels indicative of respiratory misplacement of NGT. [14]. However, drinks or drugs such as sodium bicarbonate can induce CO_2_ production in the stomach (false-positive) [15].

Our study reveals that one SNGT insertion shows airbag movement (expansion and deflation), even though the tube enters correctly into the stomach. According to Hani et al. [16], excessive air in the stomach can prevent gastric flushing in pigs used as test animals [16]. Excessive air in the stomach is caused by various factors, such as visceral hypersensitivity [17]. The contraction of abdominal muscles during expiration compresses the stomach and causes respiration-like flow in the tube [18]. Therefore, this study showed that the SNGT airbag remains static when the tip of the tube was in the esophageal tract. It has a lower sphincter that prevents the entry of air and fluids from the stomach. Thus, the second marker for the procedure should be changed from the xiphoid process to the middle part of the manubrium sterni. The trachea and esophagus are behind the manubrium sterni [19].

The results of this study did not show a significant difference in insertion accuracy between the SNGT 50% TV, SNGT 100% TV, and conventional NGT (*p* > 0.05). This is because all gastric tube insertions led to proper tube placement by senior trained staff. Chauhan et al. [20] suggested the importance of safety checks and correction by trained senior medical staff [20]. However, according to Cao et al. [21], the incidence rate of pulmonary complications caused by malpositioning of the NGT in the tracheobronchial tree branches ranges from 1.2 to 2.4%. Even with senior medical workers, malpositioning still takes place [21].

The spirometric measurements showed that the TVs of the *Macaca fascicularis* test animals were 52.8 and 50 ml. The two test animals were 4 years old and weighed 4–5 kg. The results of this study are slightly different from those of Iizuka et al. [22], who showed a TV range of 36–40 ml for 11 male *Macaca fascicularis* individuals.

The results showed a significant difference between the two types of airbags with sizes of 50% and 100% of the TV (≤ 0.05). However, the changes were an average of 3.5 mm for the SNGT 50% TV airbags and 2 mm for the SNGT 100% TV airbags. The ratio of the internal diameter is 1:3, 3 between the tube and the trachea of *Macaca fascicularis*. As a result, the air current will have difficulty entering the distal end of the tube. The tube has a diameter of 3 mm and a length of 120 cm. The tube diameter is small enough to reduce the amount of air that can pass through it, following Poiseuille's law, which states that the smaller the radius of a channel is, the greater its resistance [17]. In addition, the loss of space in the airbag that is deflated during insertion will also affect its movement. The loss of air in the equipment can interfere with the amount of gas exchange that is expected to occur [23].

The results of two pH paper tests showed a pH value of 7, which contradicts the general view that the pH of the stomach is < 7. The gastric pH of primates ranges from 2 to 6 [24]. Laboratory testing is needed to evaluate these biochemical markers. According to Metheny et al. (2017), the gastric proteolytic enzyme pepsin is a potential marker for examining tube position in the digestive tract. In a study of gastric aspiration in 32 critically ill infants, investigators used Western blot immunoassay with a sensitivity of 1 g/ml for a mean (SD) pepsin concentration of 111.9 (36.8) g/ml [25].

## Conclusion

The SNGT had high effectiveness in detecting the tube position inside the respiratory or digestive tract. An airbag with a size of 50% of the TV has better movement than that with a size of 100% of the TV. This is because of the loss of air space along the tube, which reduces the amount of air entering the tube. Therefore, the size of the airbags volume must be modified, and the procedure for marking the middle of the manubrium sterni as a second marker should be determined. In addition, we found no significant difference in insertion accuracy between the SNGT and the conventional NGT. The final verification examination of the tube position also showed that the pepsin assay was more effective than the pH paper test. This research could be advanced into human clinical trials.

## Methods

### Objectives, research model and samples

This study aimed to prove the effectiveness of the SNGT prototype in Macaca fascicularis. This research was an in vivo experimental study conducted on *Macaca fascicularis* (age: > 4 years and weight: 3–5 kg). Three animals were randomly divided into three groups as follows: the SNGT group with an airbag size of 50% of the TV, the SNGT group with an airbag size of 100% of the TV, and the conventional NGT group. The sample size was calculated using Federer's formula.

The present study was based on the replacement, reduction, and refinement (3R) principle. Therefore, three animals were used. Each animal received eight tube insertions.

### SNGT prototype

**Fig. 4:**
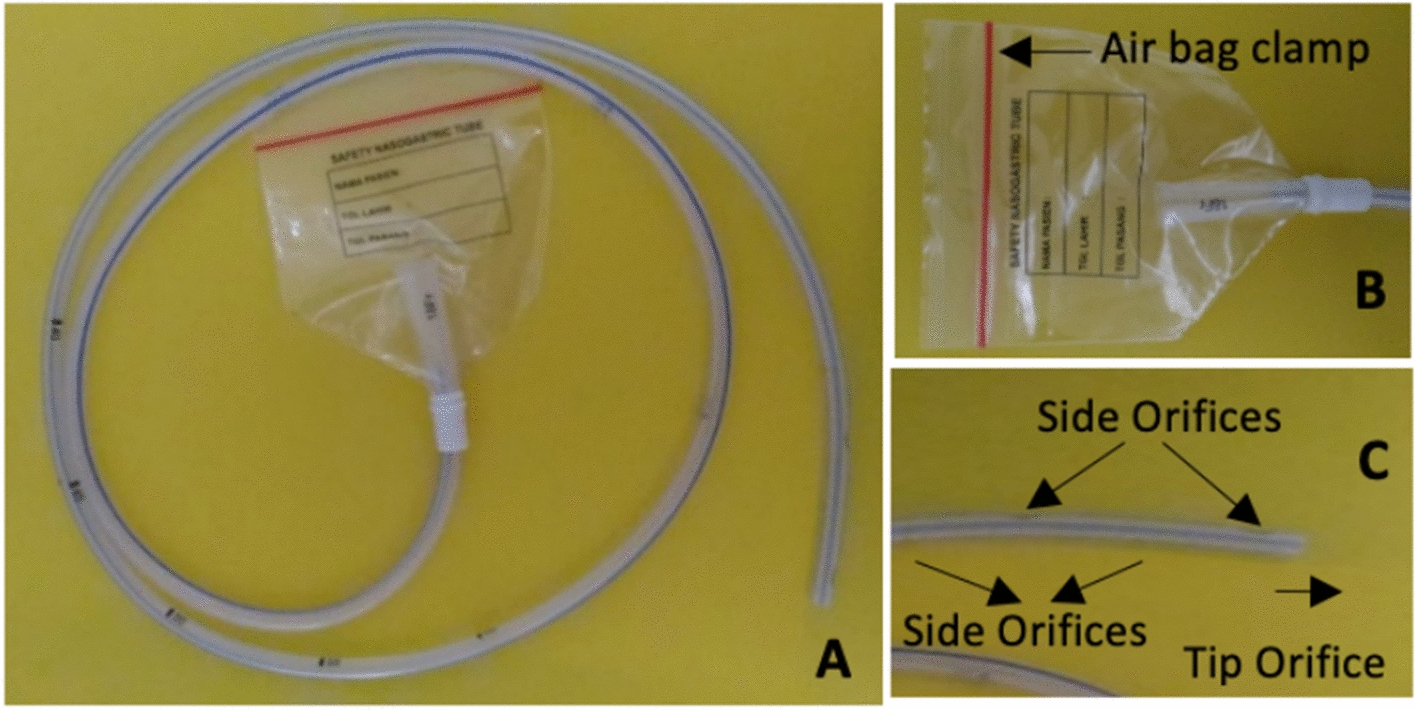
**A** Safety nasogastric tube. **B** Airbag. **C** Orifices

The prototype is shown in Fig. 4. The SNGT is equipped with an airbag at the base. The airbag has a seal that opens and closes. The seal is closed during installation to trap some air from the respiratory tract. The distal end of the SNGT has more side orifices than the traditional NGT. The number of SNGT orifices is 8–10, which is twice the common number.

Table 3 shows the differences in the NGT, SNGT, and *Macaca fascicularis* and human tracheal diameters. The size of the NGT and SNGT used was 12 Fr.

**Table 3:** Comparison of NGT, SNGT, and Macaca and human respiratory tract diameter

No	Diameter	External (mm)	Internal (mm)
1	NGT	5	3
2	SNGT	5	3
3	*Macaca fascicularis* trachea	12	10
4	Human trachea	24	20

### Procedures

#### SNGT insertion

Monkeys were fed 3 h before insertion to obtain enough 1–2 ml of gastric fluid. Monkeys were injected ketamine 10 mg/kg over a period of approximately 15 min. The monkey was placed in a semi-Fowler position with the head flexed chin-down to straighten the route to the esophagus. The tube fitting set was brought closer to the animal. The insertion length of the SNGT tube was measured external to the body, starting from the nose, ear, hyoid bone, and xiphoid process. Next, the tube was marked with tape at the boundary of the hyoid bone (first marker) and xiphoid process (second marker). The tip of the tube (15–20 cm long) was lubricated with 2% lidocaine gel, and the tube was slowly inserted through one of the monkey’s nostrils. The airbag of the SNGT moved to expand and collapse while being inserted into the upper respiratory tract. The airbag expanded during expiration and collapsed during inspiration. When the airbag expanded, a micrometer was used to measure the expansion width, which was then recorded.

The tube was inserted until it reached the limit of the first marker, after which the tape was opened. Subsequently, the tube was inserted again approximately 5 cm. If the tube passed through the esophagus, the airbag stopped moving. Conversely, the airbag moved if the tube passed into the trachea and was withdrawn. The tube was inserted again into the esophagus and advanced until it reached the second tape marker on the tube. Aspiration of stomach fluid was performed with a 10 mL syringe connected to the base of the tube, and the fluid was stored in a chemical tube at 4 °C. Then, 0.5 mL was tested with pH paper for 5 min. The SNGT tube was then removed slowly, and the monkey was returned to the cage for supervised recovery.

The stomach fluid was subsequently kept at 4 °C and then tested for pepsin activity using spectrophotometry and hemoglobin as a substrate.

#### Conventional NGT insertion

The procedure for conventional NGT insertion was similar to that for SNGT insertion but without an airbag indicator. Therefore, the marker measurement was performed only once from the nose to the earlobe and then to the xiphoid process. This marker is important for estimating the depth of insertion into the stomach.

## Data Availability

The datasets used and/or analyzed during the current study are available from the corresponding author on reasonable request.

## Abbreviations

CO_2_: Carbon dioxide
NGT: Nasogastric tube
NPSA: National Patient Safety Agency
SNGT 100% TV: SNGT with an airbag size of 100% of TV
SNGT 50% TV: SNGT with an airbag size of 50% of tidal volume
SNGT: Safety nasogastric tube
TV: Tidal volume
